# Subtypes detection of papillary thyroid cancer from methylation assay via Deep Neural Network

**DOI:** 10.1016/j.csbj.2025.04.034

**Published:** 2025-04-29

**Authors:** Andrea Colacino, Andrea Soricelli, Michele Ceccarelli, Ornella Affinito, Monica Franzese

**Affiliations:** a IRCCS SYNLAB SDN, Via Ferraris 144, Napoli, 80142, Italy; b Sylvester Comprehensive Cancer Center, Miller School of Medicine, University of Miami, 1550 N.W. 10Th Avenue, Miami, FL, 33136, USA

**Keywords:** Artificial intelligence, Deep learning, Machine learning, Convolutional neural networks, Methylome, Thyroid cancer, Papillary carcinoma subtypes

## Abstract

**Background and Objective:**

In recent years, DNA methylation-tumor classification based on artificial intelligence algorithms has led to a notable improvement in diagnostic accuracy compared to traditional machine learning methods. In cancer, the methylation pattern likely reflects both the cell of origin and somatically acquired DNA methylation changes, making this epigenetic modification an ideal tool for tumor classification. We propose an in-depth method based on the Convolutional Neural Network for the DNA methylation-based classification of papillary thyroid carcinoma (PTC) and its follicular (fvPTC) and classical (cvPTC) subtypes.

**Methods:**

To address this issue, we first performed a pan-cancer analysis to train a convolutional 1-D Neural Network (CNN) using supervised learning. Then, we evaluated the robustness of the net on an independent PTC dataset and assessed its ability to classify normal (N=56) versus tumor (N=461) samples and fvPTC (N=102) versus cvPTC (N=359). We then compared its performance with 4 machine learning models (logistic regression with elastic net penalty, quadratic discriminant analysis, support vector classifier with RBF kernel, and random forest).

**Results:**

By using RELU activation function and leaving out liquid tumors, our results show a remarkable performance of the neural network in classifying cancer and normal samples when applied to pan-cancer data (Validation AUC = 0.9903 and Validation Loss = 0.112). When applied to the thyroid independent dataset, the proposed Neural Net architecture successfully discriminates tumor versus normal samples (AUC = 0.91 +/- 0.05) and follicular versus classical PTC subtypes (AUC = 0.80 +/- 0.05), outperforming traditional machine learning algorithms.

**Conclusions:**

In conclusion, the study highlights the effectiveness of CNNs in the methylation based classification of thyroid tumors and their subtypes, demonstrating its ability to capture subtle epigenetic differences with minimal preprocessing.

This versatility makes the model adaptable for classifying other tumor types. Also, the findings emphasize the potential relevance of AI algorithms in addressing complex diagnostic challenges and supporting clinical decisions.

This research lays the foundation for developing robust and generalizable models that can advance precision oncology in cancer diagnostics.

## Graphical abstract

**Figure:**
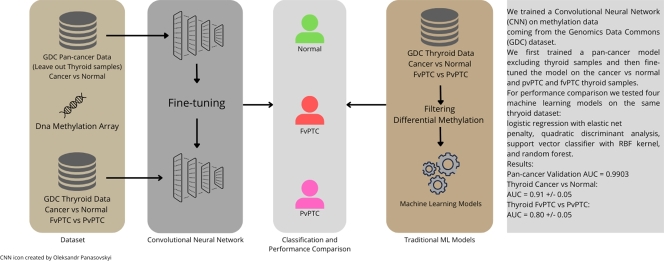

## 1 Introduction

Artificial intelligence (AI) in healthcare is reshaping the landscape of medical diagnosis, by supporting the optimization of clinical workflows and the minimization of diagnostic errors. In oncology, AI-driven predictive analytics is redefining tumor classification due to its ability to catch tumor heterogeneity and extract complex patterns from high dimensional data [1], [2], [3]. AI-driven approaches are increasingly applied in thyroid cancer diagnostics, where the differentiation between benign from malignant thyroid nodules remains a complex challenge in clinical practice [4], [5], [6].

Current diagnostic approaches rely primarily on ultrasound imaging and fine-needle aspiration cytology (FNAC), but these techniques are prone to subjective interpretation and often yield indeterminate or ambiguous results, leading to unnecessary thyroidectomies in benign cases or misclassification of aggressive subtypes [7], [8], [9], [10], [11]. To address these shortcomings, several studies have explored the integration of ultrasound imaging, histopathology data, and thyroid gland datasets with AI algorithms to improve diagnostic accuracy [8], [12], [13], [14], [15]. These AI-based tools have been employed for several tasks, including nodule classification [13], [14], [16], [17], prognosis and treatment response evaluation [18], and metastasis prediction [19]. The advent of deep learning (DL) algorithms has further enhanced AI applications in oncology, enabling automated feature extraction from raw data. Among these, Convolutional Neural Networks (CNNs) have demonstrated better performance in medical image analysis [20], [21], [22], [23], [24], [25], outperforming traditional machine learning (ML) methods through their ability to capture complex, non-linear patterns with minimal preprocessing.

While deep learning has been widely applied to imaging data, its potential in thyroid cancer DNA methylation-based classification remains to be explored. Currently, DNA methylation-tumor classification based on AI algorithms is emerging as a valuable tool for enhancing diagnostic accuracy [26], [27], [28], [29]. Aberrant methylation is a common hallmark of cancer, taking into account its initiation and progression [30]. In cancer, the methylation pattern likely reflects both the cell of origin and somatically acquired DNA methylation changes [31], [32], making this epigenetic modification an ideal tool for tumor classification [33]. Moreover, integrating deep learning methods with DNA methylation data may enable the capture of early molecular changes that precede morphological alterations visible in imaging. This makes methylation-driven analysis a valuable complement to traditional imaging methods, offering additional predictive power, especially for distinguishing between tumor subtypes and assessing prognosis.

The methylation profile of papillary thyroid carcinoma (PTC) and its subtypes can provide a molecular signature for a best classification. However, existing methods primarily use traditional ML approaches. For example, [34] applied an unsupervised machine learning method to classify different follicular cell-derived thyroid neoplasms exclusively on their DNA methylation pattern. Despite these advancements, no study to date has leveraged deep learning-based models for robust, methylome-driven classification of PTC and its subtypes. Given the diagnostic challenges to distinguish follicular variant PTC (fvPTC) from classical PTC (cvPTC), a more accurate, AI-driven molecular classification approach is needed.

**Fig. 1:**
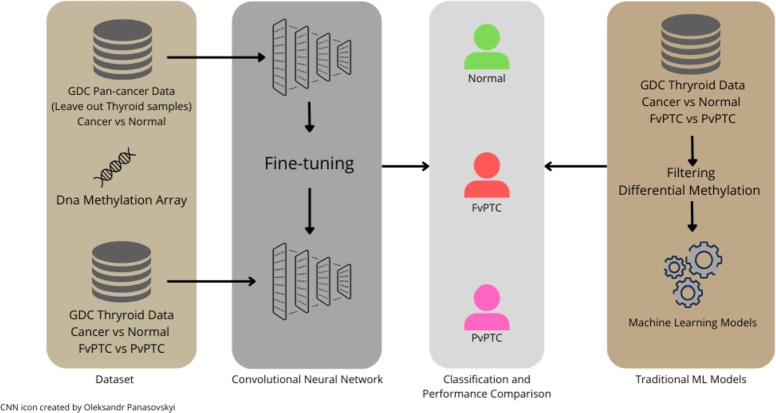
High-level workflow.

To address this gap, we present a Convolutional Neural Network (CNN)-based framework for the classification of PTC and its subtypes (fvPTC and cvPTC) using DNA methylation data. For this purpose, we first evaluated the diagnostic performance of traditional ML algorithms (Logistic Regression, Random Forest, Quadratic Discriminant Analysis) on methylation data. Then, we trained a 1D CNN model using supervised learning on a Pan-Cancer DNA methylation dataset and cross-validated it using Repeated Stratified 5-fold cross-validation on 461 PTC patients (102 fvPTC and 359 cvPTC) and 56 normal samples. Finally, we assessed the performance of all models after applying the differential methylation analysis (dmCpGs) as a variable selection technique, reducing the dimensionality since many Machine Learning Models fail to handle the incredibly large number of features produced by methylation arrays. The differential analysis step is commonly integrated into the data preprocessing workflow to reduce the dimensionality of high-throughput data, minimize the noise and to refine feature selection for ML and DL models ([35], [36], [37]). By integrating deep learning with epigenomic data, this study provides a highly reproducible, clinically relevant tool that could refine PTC classification and its subtype, reduce misdiagnosis, and support precision medicine strategies in thyroid cancer management. The workflow of our approach is summarized in Fig. 1.

## 2 Materials and methods

### 2.1 Data source and processing

Methylation data were retrieved from the Genomic Data Commons (GDC) data portal (https://portal.gdc.cancer.gov/). To train the Neural Network, we performed a pan-cancer analysis by using all the tumor types stored in the GDC portal corresponding to 9965 non-thyroid cases. For this analysis, we excluded tumors with less than 10 total cases (normal and tumors), whose methylation data were obtained by 27k Illumina Infinium Methylation Beadchip, and thyroid cancer.

Missing probes (NAs) were handled with zero-imputation, since for deep neural nets zeroes that for 1-D convolutional layers work similarly to masking in 2D. The GDC manifest for the relevant cases is available in section 6. Thyroid cancer cases (461 PTC, 56 Solid Tissue Normal) were used to evaluate the performance of traditional machine learning models (Logistic Regression, Quadratic Discriminant Analysis, Support Vector Classifier with RBF kernel, and Random Forest classifier) and for testing Neural Network. Specifically, we tested two conditions: unfiltered (485'577 CpGs) and filtered (394'337 CpGs) datasets.

In the filtered dataset, where technical and gender-specific biases have been excluded, we factored out:

1. probes whose methylation value was reported in less than 90% of samples in each group (PTC and normal);
2. uncommon probes among the three groups;
3. probes associated with sexual chromosomes and SNPs;
4. probes containing CHG or CHH;
5. and cross-reactive probes [38].

Missing values have been imputed using median imputation.

When feeding the filtered dataset to the Neural network models, we adopted a strategy borrowed from image analysis. Specifically, given the different number of CpGs between the filtered dataset and those given as input to the net, to evaluate its performance, we put the filtered features to zero, like zero-padding images for standard 2D conv-nets for restoring training dimensionality.

### 2.2 Machine learning and deep learning classification models

In this work, we compared the performance of traditional machine-learning models widely used in the literature 2.2.2 with a convolutional deep learning approach 2.2.1 in discriminating histologically defined PTC patients vs normal tissue and PTC subtypes (cvPTC vs fvPTC).

#### 2.2.1 Deep learning model: architecture and training on GDC pan-cancer dataset

**Fig. 2:**
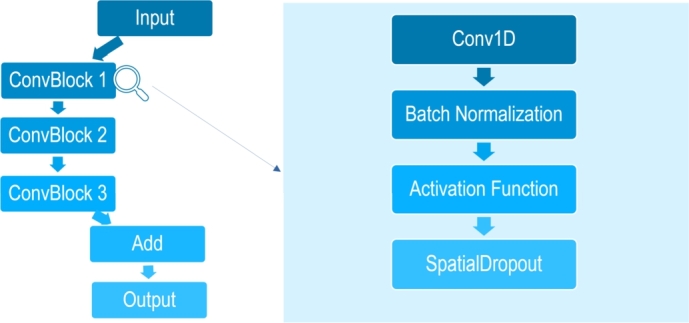
Graphical Depiction of Neural Network Architecture.

We implemented a 1D-convolutional neural network (1D-CNN) for classification, following the architecture illustrated in Fig. 2. The choice of a 1D-CNN was motivated to exploit the sequential nature of methylation array data. 1D-CNN is also employed for temporal sequence data and are producing state-of-art solutions for domains where data is monodimensional [39]. 1D-CNN has been successfully used in bioinformatics contexts, such as classification with gene expression microarrays [40]. The dimensionality of the first layer was set to 485577 matching the methylation array data size.

The network consists of three Residual Convolutional blocks with 16,32 and 64 filters respectively. Each block in interleaved with an AveragePooling1D layer. Within each block, a 1D-convolutional layer is followed by one or two residual units, each comprising a 1D-convolutional layer, batch normalization, an activation function, and SpatialDropout1D. The first convolutional block contains one residual unit, while the second and the third blocks contain each one two residual units.

After the last convolutional block, there is a flatten layer and a dense layer with a sigmoid activation function for classification. The use of residual convolutional blocks was chosen to avoid the vanishing gradient problem. Additionally, the inclusion of dropout and batch normalization aims to increase the robustness of the net towards outlier data, improve the generalization capabilities and reduce training time.

Two activation functions (LeakyReLU and standard ReLU) were tested during training. The training on the pan-cancer dataset lasted for 30 epochs, with a validation set comprising 20% of the original data, selected using stratified sampling based on the outcome label (cancer vs. normal). The model was trained using binary cross-entropy as the loss function and the Adam optimizer algorithm with default parameters to update the weights of network iterative based on training data. A loss function is used in Machine Learning and Deep Learning for quantifying the difference between the model's predicted output and the actual target value. The definition of Epoch is a complete pass through the entire training set, the number of epochs thus represents the amount of times a model has seen each instance in the dataset.

The training process was performed with early stopping after 10 consecutive epochs without improvement in validation ROC AUC. This process, known as early-stopping, avoids over-fitting of the model on the training set and cuts unnecessary training time. Additionally, a learning rate reduction was applied on loss plateaus after 5 epochs. Model performance was evaluated through error analysis, and misclassification patterns were investigated.

The network was initially trained on a pan-cancer dataset (both tumor and normal samples) and subsequently fine-tuned specifically on thyroid samples for two classification tasks: distinguishing PTC from normal samples, thus considering cvPTC and fvPTC as a whole, and differentiating between the two PTC variants, cvPTC vs fvPTC.

By combining the prediction of the cancer and the subtype CNN models, we obtained an end-to-end classification framework able to distinguish normal, fvPTC and cvPTC samples. Further details on performance evaluation and optimization strategies are provided in the Section 2. Additionally, for a complete reproducible example of all analysis together with the trained models see Section 6. The network was compiled and trained using Keras 2.14.0.

The validation phase has been performed as reported in 2.3. For the thyroid cancer task, fine-tuning the CNN consisted in resuming the training on the thyroid samples only, reducing the learning rate to avoid disruption of learned weights. For sub-type classification task the output layer was reset with random weight before re-training on cvPTC vs fvPTC. Both models were trained using K-fold Cross-Validation as detailed in section 2.3.

We trained all CNN models using an Nvidia QUADRO RTX 5000 GPU. Details regarding model weights and the complete code implementation are provided in Section 6.

#### 2.2.2 Traditional machine learning algorithms

Four different machine-learning approaches widely used in methylation classification studies [41], [42], [43] were trained to classify both PTC versus normal tissue: Logistic Regression (LR) with the elastic-net penalty, Quadratic Discriminant Analysis (QDA), Support Vector Classifier (SVC) with RBF kernel, and Random Forest (RF). LR is an extension of linear regression which predicts the probability that an instance belongs to a given class [44]. QDA is a variant of Linear discriminant analysis (LDA) that allows for nonlinear separation of data [45]. SVC belongs to a general field of kernel-based machine learning methods and is used to classify both linearly and non-linearly separable data [46]. RF is an ensemble learning method for classification that operates by constructing a multitude of decision trees during training and outputting the class that is the mode of the classes (classification) [47], [48] We tested an additional complex ensemble model, LightGBM, on differential methylation data due to the difficulties of this model in handling the very high-dimensionality data of methylation array.

Machine learning methods were trained with the following parameters:

1. Logistic Regression: penalty='elasticnet',C=0.02,l1_ratio=0.7;
2. QDA: default parameters after variable selection from Logistic Regression;
3. SVC: C = 0.02;
4. RandomForest: n_estimators=50, max_depth=3, max_features=log2.

All models have been trained with balanced class weights to account for imbalance in the dataset. The training has been performed using scikit-learn: 1.3.2. The validation phase details are reported in section 2.3.

### 2.3 Validation phase for machine learning and deep learning models

In the training phase of Machine Learning and Deep Learning models, we used 80% of GDC dataset for training set and the remaining 20% for validation. To robustly evaluate the performance on all models (Machine Learning and Deep Learning) on the thyroid dataset without sacrificing many samples, we employed a Repeated Stratified k-fold Cross-Validation (CV), with k=5 and repeated 5 times. When using stratified k-fold CV, the dataset is splitted in 5 parts, thus ensuring that the proportion of target variables is equal in all subsets: k−1 folds form the training set and the remaining fold constitutes the test set. In the case of Repeated K-fold the entire process is iterated and the results are averaged to ensure a robust estimate of the generalization error. The target variable was cancer presence for the cancer classification task and PTC subtype for the subtype task. All models, both traditional ML and CNN, were tested on the same folds and then the result for each model is averaged. This ensures comparison on the same data.

### 2.4 Evaluation of machine learning and deep learning model performance

The chosen metrics performance were Accuracy, Precision, Recall, F-Measure, and ROC AUC. When evaluating a classification model, the samples identified by the model can be divided in four classes:

• True Positives (TP): Positive samples correctly identified as positives;
• True Negatives (TN): Negative samples correctly identified as negatives;
• False Positives (FP): Negative samples wrongly identified as positives;
• False Negatives (FN): Positive samples wrongly identified as negatives.

A compact way for visualizing the performance is the confusion matrix, see Fig. 4 for an example.

Accuracy is defined as the number of correctly identified instances divided by the total amount of sample or equivalently:

$$\frac{TP+TN}{TP+TN+FP+FN}$$

Since the thyroid cancer dataset is imbalanced (Positives: 102, Negatives: 359), it is preferable to use a different metric or evaluate the performance of a dummy classifier for the baseline (e.g. a classifier that always outputs the majority class or with probability density equal to the dataset distribution). We employed both approaches and reported the metrics compared to the baseline dummy estimation and we included better suited metrics like precision, recall, and ROC-AUC.

Precision is defined as the number of true positives divided by the total number of samples predicted as positives:

$$\frac{TP}{TP+FP}$$

Recall is defined as the number of true positives divided by the total number of positive samples in the dataset:

$$\frac{TP}{TP+FN}$$

F-measure is defined as the harmonic mean of Precision and Recall:

$$2⁎\frac{precision⁎recall}{precision+recall}$$

ROC curve is defined as the plot of TPF (sensitivity) versus FPF (1-specificity) using different thresholds. AUC is a combined measure of sensitivity and specificity and describes the entire curve rather than depending on the specific point [49].

### 2.5 Differential methylation-based feature selection

To verify whether a preliminary differential methylation-based feature selection could impact the performance of ML and DL models, we first identified differentially methylated CpG sites (dmCpGs) and subsequently used them as input features for classification tasks. Differential methylation occurs when CpG sites exhibit significant differences in methylation levels between distinct experimental conditions, thus potentially contributing to disease phenotype [50], [51], [30]).

To detect dmCpGs, we performed a differential methylation analysis comparing Tumor vs Normal and cvFTC vs fvPTC using the limma R/Bioconductor package ([52]). Differential methylation was assessed based on log2 fold change (logFC), which quantifies the extent of methylation differences between experimental conditions, and false discovery rate (FDR), which controls for multiple comparisons when multiple hypotheses are being tested at once. CpG sites were considered as differentially methylated if they exhibited |logFC|⩾1 and an FDR⩽0.05.

## 3 Results

### 3.1 Pan-cancer training of neural net

**Fig. 3:**
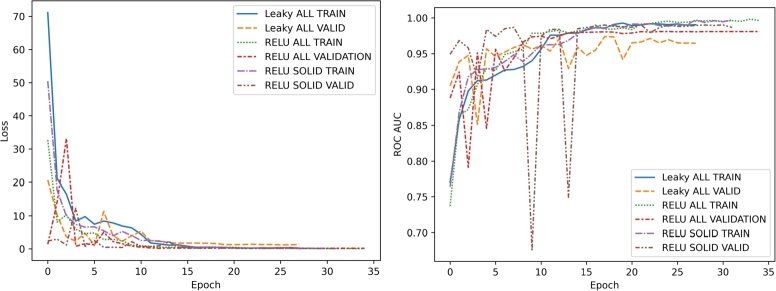
Comparison of training and validation performance across epochs for different activation functions in pan-cancer analysis. Line plots display the evolution of binary cross-entropy loss (left) and ROC AUC (right) over 30 training epochs for 1D-CNN models utilizing either LeakyReLU or standard ReLU activation functions. Models were trained on a pan-cancer dataset including both solid and liquid tumors, as well as on a refined subset containing only solid tumors. Specifically, the following models were trained and validated: i) 1D-CNN using LeakyReLU on the complete dataset (Leaky ALL); 2) 1D-CNN using standard ReLU on the complete dataset (ReLU ALL); 3) 1D-CNN using standard ReLU trained solely on solid tumors (ReLU SOLID). Among all tested configurations, the model trained exclusively on solid tumors using the standard ReLU activation function yielded the most accurate performance, as shown by lower validation loss and higher validation ROC AUC values. In particular, the best-performing model weights were obtained at epoch 22 (Validation AUC = 0.9903, Validation Loss = 0.112). Line styles indicate the data partition: solid lines represent the training set, while dashed lines correspond to the validation set.

As a first step in training our 1D-CNN, we tested both LeakyReLU and standard ReLU activation functions on the pan-cancer dataset composed of 9131 tumors (both solid and liquid) and 829 normal samples to evaluate their impact on model performance and identify the most effective one. Performance metrics for each of the 30 training epochs, including both training and validation sets, are summarized in Fig. 3 and detailed in the Supplementary Table 1, Table 2. As shown in Fig. 3 and the Supplementary Table 1, Table 2, the model using standard ReLU outperformed the one using LeakyReLU in terms of both ROC AUC and binary cross-entropy loss (Standard ReLU: Validation AUC = 0.981, Validation Loss = 0.1651; LeakyReLU: Validation AUC = 0.9739, Validation Loss = 1.271).

**Table 1:** Comparison of Normal vs Cancer Classification Performance using unfiltered probes (positive = fvPTC or cvPTC, negative = normal).

	Accuracy	AUC ROC	Recall Neg	Recall Pos	Precision Neg	Precision Pos	F Neg	F Pos
Dummy	0.808+/-0.035	0.50+/-0.06	0.11+/-0.11	0.893+/-0.035	0.11+/-0.10	0.892+/-0.013	0.11+/-0.10	0.892+/-0.021
NeuralNetwork	0.92+/-0.04	0.91+/-0.05	0.89+/-0.10	0.92+/-0.04	0.61+/-0.13	0.986+/-0.012	0.72+/-0.10	0.953+/-0.024
Logistic-elastic	0.820+/-0.031	0.899+/-0.018	1.0+/-0	0.798+/-0.035	0.38+/-0.04	1.0+/-0	0.55+/-0.04	0.887+/-0.022
QDA	0.81+/-0.04	0.56+/-0.08	0.25+/-0.15	0.88+/-0.04	0.20+/-0.12	0.905+/-0.018	0.22+/-0.13	0.890+/-0.024
RF	0.951+/-0.017	0.78+/-0.08	0.57+/-0.15	0.998+/-0.004	0.98+/-0.05	0.950+/-0.017	0.70+/-0.13	0.973+/-0.009
SVC	0.69+/-0.05	0.826+/-0.025	0.996+/-0.018	0.65+/-0.05	0.263+/-0.031	0.9994+/-0.0029	0.41+/-0.04	0.79+/-0.04

**Table 2:** Comparison of Normal vs Cancer Classification Performance using filtered probes (positive = fvPTC or cvPTC, negative = normal).

	Accuracy	AUC ROC	Recall Neg	Recall Pos	Precision Neg	Precision Pos	F Neg	F Pos
Dummy	0.806+/-0.030	0.49+/-0.04	0.08+/-0.09	0.894+/-0.034	0.08+/-0.08	0.889+/-0.011	0.08+/-0.08	0.891+/-0.019
NeuralNetwork	0.949+/-0.023	0.88+/-0.06	0.79+/-0.13	0.968+/-0.030	0.79+/-0.14	0.975+/-0.015	0.77+/-0.08	0.971+/-0.014
Logistic-elastic	0.809+/-0.032	0.893+/-0.018	1.0+/-0	0.79+/-0.04	0.37+/-0.04	1.0+/-0	0.53+/-0.04	0.880+/-0.023
QDA	0.78+/-0.05	0.54+/-0.09	0.24+/-0.16	0.84+/-0.05	0.17+/-0.13	0.901+/-0.021	0.19+/-0.14	0.871+/-0.033
RF	0.947+/-0.017	0.76+/-0.08	0.52+/-0.16	0.998+/-0.004	0.98+/-0.05	0.945+/-0.018	0.66+/-0.14	0.971+/-0.009
SVC	0.68+/-0.05	0.816+/-0.026	0.996+/-0.018	0.64+/-0.06	0.253+/-0.030	0.9994+/-0.0029	0.40+/-0.04	0.78+/-0.04

**Figure S1:**
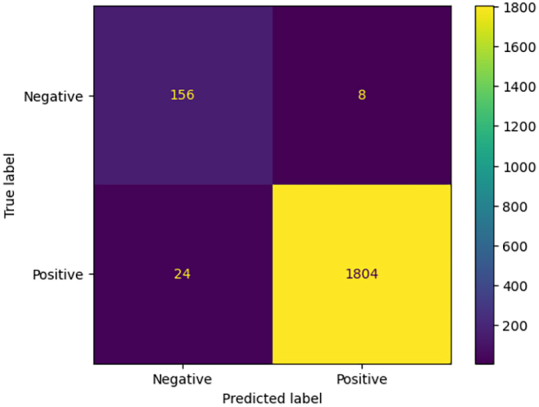
Confusion matrix of CNN on validation set when using all non-thyroid samples.

Despite the overall good performance, both activation functions yielded a high false negative rate, primarily involving samples from liquid tumors (acute myeloid leukemia, AML). A detailed performance breakdown of the pan-cancer model for each study in the GDC portal is available in the Supplementary Fig. 1.

**Fig. 4:**
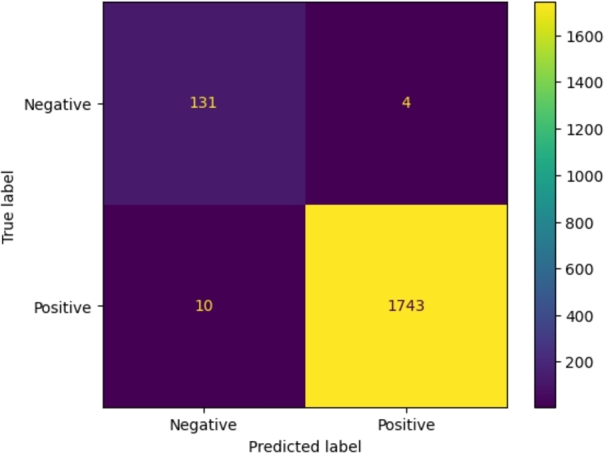
Confusion matrix of neural network trained on solid tumors with ReLU activation function.

Based on these findings, we retrained our 1D-CNN with standard ReLU activation function and by excluding liquid tumors, thus resulting in a dataset of 8,760 solid tumors and 788 normal samples (training + validation). During this training phase, the model achieved its best performance (Validation AUC = 0.9903 and Validation Loss = 0.112) at epoch 22 and a marked reduction in false negatives. As shown in the validation set confusion matrix reported in Fig. 4, the model demonstrated remarkable classification performance, properly identifying most of tumor and normal samples. Specifically, it misclassified only 10 tumor samples as normal (false negatives) and 4 normal samples as tumors (false positives), supporting the effectiveness of the model in distinguishing solid tumors from normal tissue.

### 3.2 Normal vs tumor patients classification

We fine-tuned the net on the thyroid dataset for cancer (461 pz) vs normal (56 pz) detection. Both for machine learning and neural network algorithms, we tested two conditions: 1) unfiltered (485'577 CpGs) and filtered (394'337 CpGs) datasets. See Section 2 for filtering details.

The Neural Network and Machine learning algorithms showed notable differences in diagnostic performance depending on the dataset under consideration. Of all models, Neural Network showed the best performance (ROC AUC = 0.91+/-0.05) on unfiltered data, while logistic regression performed better on filtered data (ROC AUC = 0.893+/-0.018).

### 3.3 fvPTC vs cvPTC patients classification

We then fine-tuned the net on the thyroid dataset for cancer subtype detection, distinguishing between the follicular variant subtype (fvPTC, 102pz) and the classic variant subtype (cvPTC, 359 pz). The net was able to learn features during the pancancer training useful enough to distinguish between subtypes with minimal retraining.

Out of all models, Neural Network showed the best performance (ROC AUC = 0.77+/-0.06) on unfiltered data, and it was still the best on filtered data (ROC AUC = 0.78+/-0.04). Although the net performance decreased from the classification task, it is notably the best model for both tasks with comparable performance when using filtered or unfiltered probes. See Table 3, Table 4

**Table 3:** Comparison of fvPTC vs cvPTC Classification Performance using unfiltered probes (positive = fvPTC, negative = cvPTC).

	Accuracy	AUC ROC	Recall Neg	Recall Pos	Precision Neg	Precision Pos	F Neg	F Pos
Dummy	0.654+/-0.035	0.50+/-0.05	0.78+/-0.04	0.22+/-0.10	0.778+/-0.024	0.21+/-0.08	0.777+/-0.025	0.21+/-0.08
NeuralNetwork	0.83+/-0.04	0.77+/-0.06	0.87+/-0.04	0.66+/-0.11	0.901+/-0.027	0.60+/-0.08	0.886+/-0.026	0.62+/-0.08
Logistic-elastic	0.230+/-0.008	0.505+/-0.006	0.012+/-0.009	0.998+/-0.010	0.7+/-0.5	0.223+/-0.005	0.023+/-0.017	0.364+/-0.008
QDA	0.62+/-0.06	0.51+/-0.06	0.72+/-0.07	0.30+/-0.09	0.782+/-0.027	0.24+/-0.08	0.75+/-0.05	0.26+/-0.08
RF	0.783+/-0.020	0.527+/-0.032	0.986+/-0.017	0.07+/-0.06	0.789+/-0.014	0.5+/-0.4	0.876+/-0.012	0.12+/-0.10
SVC	0.799+/-0.027	0.62+/-0.05	0.941+/-0.032	0.30+/-0.10	0.826+/-0.019	0.60+/-0.14	0.879+/-0.017	0.39+/-0.11

**Table 4:** Comparison of fvPTC vs cvPTC Classification Performance using filtered probes (positive = fvPTC, negative = cvPTC).

	Accuracy	AUC ROC	Recall Neg	Recall Pos	Precision Neg	Precision Pos	F Neg	F Pos
Dummy	0.644+/-0.035	0.49+/-0.05	0.76+/-0.04	0.22+/-0.10	0.775+/-0.023	0.21+/-0.08	0.769+/-0.024	0.21+/-0.09
NeuralNetwork	0.830+/-0.030	0.78+/-0.04	0.87+/-0.04	0.70+/-0.09	0.911+/-0.024	0.61+/-0.07	0.888+/-0.021	0.65+/-0.06
Logistic-elastic	0.237+/-0.017	0.509+/-0.013	0.021+/-0.020	0.996+/-0.014	0.7+/-0.4	0.224+/-0.007	0.04+/-0.04	0.366+/-0.010
QDA	0.62+/-0.05	0.52+/-0.06	0.70+/-0.06	0.34+/-0.10	0.790+/-0.032	0.25+/-0.06	0.74+/-0.04	0.29+/-0.07
RF	0.787+/-0.017	0.538+/-0.033	0.986+/-0.013	0.09+/-0.06	0.792+/-0.012	0.6+/-0.4	0.878+/-0.009	0.15+/-0.10
SVC	0.803+/-0.024	0.63+/-0.04	0.939+/-0.033	0.32+/-0.10	0.831+/-0.017	0.62+/-0.11	0.881+/-0.016	0.42+/-0.09

### 3.4 Differential methylation: normal vs cancer

To assess whether selecting epigenetically altered CpGs improves model accuracy or yields comparable predictive power, we performed a preliminary differential methylation-based feature selection. The identified dmCpGs were then used as input features for ML and DL models to evaluate their impact on classification performance.

In the Tumor vs Normal comparison, we identified N = 10899 dmCpGs. By comparing the classification accuracy of ML and DL models trained on the full set of CpGs Table 1, Table 2 versus only dmCpGs Table 5, we observed that reducing the number of irrelevant variables – and consequently the noise – enhanced the performance of all models. Notably, despite training the CNN zero-padding the non-differentially methylated probes (See Section 2.5, the results are comparable to those of other ML methods and its performance were even consistent with that obtained on the unfiltered dataset. Specifically, as reported in Table 5 the CNN achieved the highest AUC ROC (0.969+/-0.025) followed by LightGBM (0.96+/-0.011).

**Table 5:** Comparison of Normal vs Cancer Classification Performance using differentially methylated probes (positive = fvPTC or cvPTC, negative = normal).

	Accuracy	AUC ROC	Recall Neg	Recall Pos	Precision Neg	Precision Pos	F Neg	F Pos
Dummy	0.805+/-0.032	0.50+/-0.05	0.12+/-0.08	0.889+/-0.033	0.12+/-0.10	0.892+/-0.010	0.12+/-0.08	0.890+/-0.019
NeuralNetwork	0.972+/-0.015	0.969+/-0.025	0.96+/-0.05	0.973+/-0.017	0.83+/-0.09	0.996+/-0.006	0.89+/-0.06	0.984+/-0.008
Logistic-elastic	0.84+/-0.04	0.905+/-0.019	0.993+/-0.025	0.82+/-0.04	0.40+/-0.05	0.9990+/-0.0034	0.57+/-0.05	0.898+/-0.025
QDA	0.79+/-0.07	0.59+/-0.07	0.32+/-0.10	0.85+/-0.07	0.24+/-0.14	0.911+/-0.015	0.26+/-0.10	0.88+/-0.04
RF	0.982+/-0.013	0.92+/-0.06	0.85+/-0.12	0.998+/-0.004	0.98+/-0.04	0.982+/-0.014	0.90+/-0.08	0.990+/-0.007
SVC	0.955+/-0.021	0.959+/-0.026	0.96+/-0.05	0.954+/-0.024	0.73+/-0.10	0.996+/-0.006	0.83+/-0.07	0.974+/-0.012
LGBM	0.986+/-0.011	0.96+/-0.04	0.92+/-0.08	0.994+/-0.007	0.95+/-0.06	0.991+/-0.010	0.93+/-0.06	0.992+/-0.006

### 3.5 Differential methylation: fvPTC vs cvPTC

In the cvPTC vs fvPTC comparison, we identified N = 5281 dmCpGs. As reported in Table 6, the CNN achieved the best AUC ROC (0.80 ± 0.05), comparable to the performance of the SVC model. However, compared to SVC, the CNN demonstrated slightly higher accuracy and F-measure.

**Table 6:** Comparison of fvPTC vs cvPTC Classification Performance using differentially methylated probes (positive = fvPTC, negative = cvPTC).

	Accuracy	AUC ROC	Recall Neg	Recall Pos	Precision Neg	Precision Pos	F Neg	F Pos
Dummy	0.667+/-0.034	0.504+/-0.033	0.80+/-0.05	0.21+/-0.07	0.781+/-0.015	0.23+/-0.06	0.788+/-0.027	0.22+/-0.06
NeuralNetwork	0.83+/-0.04	0.80+/-0.05	0.85+/-0.06	0.74+/-0.11	0.922+/-0.027	0.60+/-0.09	0.883+/-0.033	0.66+/-0.07
Logistic-elastic	0.78+/-0.04	0.78+/-0.05	0.78+/-0.05	0.78+/-0.09	0.928+/-0.029	0.51+/-0.06	0.848+/-0.031	0.62+/-0.06
QDA	0.60+/-0.06	0.54+/-0.07	0.65+/-0.06	0.43+/-0.13	0.80+/-0.04	0.26+/-0.07	0.71+/-0.05	0.32+/-0.08
RF	0.824+/-0.032	0.73+/-0.05	0.90+/-0.04	0.56+/-0.10	0.878+/-0.026	0.62+/-0.08	0.888+/-0.022	0.58+/-0.07
SVC	0.81+/-0.04	0.80+/-0.05	0.82+/-0.04	0.77+/-0.08	0.927+/-0.026	0.56+/-0.07	0.870+/-0.031	0.64+/-0.07
LGBM	0.82+/-0.04	0.71+/-0.05	0.902+/-0.035	0.51+/-0.09	0.867+/-0.024	0.61+/-0.10	0.884+/-0.025	0.55+/-0.09

## 4 Discussion

Accurate classification of PTC and its subtypes is crucial for guiding treatment decisions. Current clinical diagnosis primarily relies on morphological assessment and subjective interpretation through ultrasound imaging and fine-needle aspiration cytology (FNAC). However, these methods often yield indeterminate or ambiguous results, leading to unnecessary thyroidectomies in benign cases or misclassification of aggressive subtypes. These challenges highlight the need for alternative approaches and DNA methylation is emerging as a complementary strategy for refining thyroid carcinoma classification ([53], [54], [55], [56]). While traditional machine learning (ML) models have been employed for DNA methylation-based classification of PTC and its subtypes [34], they often face limitations due to the need for extensive preprocessing and dimensionality reduction, which can lead to information loss and limit their ability to fully capture the intra-tumoral heterogeneity of PTC [57]. In contrast, convolutional neural networks (CNNs) have been widely applied in thyroid cancer classification, primarily leveraging imaging data to support clinical diagnosis [15]. However, their application to molecular data, such as DNA methylation, remains less explored. Our study extends the use of CNNs beyond imaging by employing them for methylation-based classification of PTC and its subtypes (fvPTC and cvPTC). AI-driven approach provides an objective, data-driven molecular classification and allows for the identification of complex or nuanced methylation patterns that may not be captured by traditional ML models or conventional diagnostic tools ([58], [59], [60]). The main results are:

1. the CNN shows high predictive performance in pan-cancer classification;
2. the CNN outperforms ML methods while bypassing extensive pre-processing;
3. the CNN trained on unfiltered data achieves consistent performance even when non-dmCpGs probes are filtered out from the input.

In line with [61], our results show the remarkable predictive performance of the neural network (NN) in classifying cancer and normal samples in the pan-cancer analysis. Indeed, despite the inherent heterogeneity of molecular and clinical profiles, by employing the standard ReLU activation function and refining the dataset through the exclusion of liquid tumor data, the NN achieved an AUC of 0.9903. This approach also led to a significant reduction of false positive and false negative, a crucial aspect in clinical applications [62]. Consistent with [63], our results confirm the effectiveness of deep learning in cancer classification using DNA methylation, underscoring the critical importance of dataset refinement and architectural optimization, particularly in the domains of data preprocessing, feature selection, and model design. Specifically, the DNN pipeline prioritizes unsupervised dimensionality reduction and pan-cancer scalability, achieving a maximum AUC ROC of 0.89. In contrast, the CNN-based approach incorporates biologically informed feature filtering and activation function optimization, reaching an AUC ROC of 0.99 on solid tumors, using a ReLU model. Moreover, our study extends to cancer subtype discrimination (cvPTC vs. fvPTC) achieving the highest AUC ROC (0.80 ± 0.05) with minimal retraining, demonstrating that representations learned from pan-cancer data can generalize well to intra-cancer heterogeneity.

The robustness of the CNN in handling the complex and high-dimensional DNA methylation data is supported by its reliable performance on an independent thyroid dataset. Indeed, compared to traditional ML models, our CNN exhibited superior classification ability despite data noise and missing values. Specifically, our CNN model achieved an AUC of 0.91 ± 0.05 on unfiltered data for tumor versus normal classification and an AUC of 0.80 ± 0.05 on both filtered and unfiltered data for PTC subtypes (fvPTC and cvPTC) discrimination, outperforming ML algorithms that struggled to capture subtle methylation differences. Notably, logistic regression with elastic net penalty, despite its efficiency and widespread use in epigenetic research (([64], [65], [66], [67], [68]), failed to capture complex and non-linear interactions among features [69] as effectively as CNN. Moreover, the CNN performance on dmCpGs remained consistent with that obtained on the full dataset, highlighting its ability to capture key methylation patterns even in the presence of noisy or redundant features. The CNN's ability to bypass extensive preprocessing [70], [71]), reduces the dependency on condition-specific adjustments, promoting its adaptability for clinical use. Our findings are consistent with those reported by [15], confirming that CNNs achieve sensitivity, specificity, and accuracy comparable to or even superior to those of experienced radiologists. In line with previous studies, they also observed that CNN-assisted diagnosis significantly improves the performance of less experienced radiologists, enhancing both sensitivity and overall classification accuracy. Additionally, we acknowledge that hybrid approaches combining CNNs with other machine learning techniques could further refine diagnostic performance, as highlighted in the literature ([15]).

Despite these strengths, our study has certain limitations. First, the absence of an external validation cohort for PTC poses a challenge in assessing the generalizability of our model. External validation is essential to confirm robustness across independent datasets and minimize concerns regarding potential overfitting. Future studies should integrate additional independent datasets to further refine the model's predictive performance and enhance its applicability in clinical settings. Second, the GDC dataset exhibits an imbalance favoring cancer samples over normal samples, which may introduce bias by skewing classification performance toward the majority class during training and reduce sensitivity in detecting normal samples. Addressing this issue in future work could further improve diagnostic sensitivity and specificity.

Furthermore, while our CNN model exhibits promising classification performance, integrating additional multi-omics data (e.g., transcriptomics, proteomics) could further refine and enhance diagnostic precision by capturing a more comprehensive molecular landscape. Future studies could explore multimodal approaches to strengthen the model's predictive power and broaden the applicability of AI-driven classification models in oncology.

Despite these limitations, our study highlights the potential of deep learning for methylation-based classification of PTC and its subtypes, reinforcing the importance of AI-driven approaches in addressing complex diagnostic challenges in precision oncology. By addressing current limitations, this study may lay the groundwork to develop even more reliable and generalizable AI-assisted cancer diagnostic tools that can complement traditional clinical assessments and enhance diagnostic accuracy.

## 5 Conclusions

This work highlighted the potential of deep learning for methylation-based classification of thyroid carcinoma. Specifically, the use of methylation in a convolutional neural net is promising for distinguishing subtypes that are difficult to identify early, possibly allowing diagnosis with an effective and time-efficient technique that will benefit subsequent research on the topic. By leveraging high-dimensional data and addressing computational challenges, our approach could represent a promising breakthrough in precision oncology. The model can be reused for other work thus amortizing the computational cost of pan-cancer learning on several tasks. It is also worth pointing out that the retraining phase is much less time and resource-intensive than full training especially when GPU resources are available. Potential future developments may include extending the application of this model to encompass other tumor types, utilizing larger and more diverse cohorts to enhance its generalizability and integrating additional molecular data such as transcriptomic or proteomic profiles. This approach could further improve the model's utility to capture uncovering complex biological patterns and identify clinically actionable biomarkers with more precision.

## 6 Availability of software

Refer to the Github repository and Zenodo for a complete reproducible example of all analysis together with the trained models. All the code for downloading the relevant cases from the GDC portal and reproducing the code is available at the following GitHub repository: https://shorturl.at/gdgNV. See Zenodo for the GDC thyroid dataset and the learned models: https://shorturl.at/f86nd

## CRediT authorship contribution statement

**Andrea Colacino:** Writing – original draft, Visualization, Validation, Software, Methodology, Data curation. **Andrea Soricelli:** Writing – review & editing, Supervision. **Michele Ceccarelli:** Writing – review & editing, Supervision, Methodology. **Ornella Affinito:** Writing – original draft, Supervision, Methodology, Formal analysis, Data curation. **Monica Franzese:** Writing – review & editing, Supervision, Methodology, Formal analysis, Conceptualization.

## Declaration of Competing Interest

The authors declare that there are no conflicts of interest relevant to this study. All funding sources for the research were appropriately disclosed, and there were no external influences on the study design, data analysis, or interpretation. The authors have no financial or personal relationships that could have influenced the research outcome. This ensures the transparency and integrity of the work presented. Andrea Colacino

## Figures and Tables

**Figure S2:**
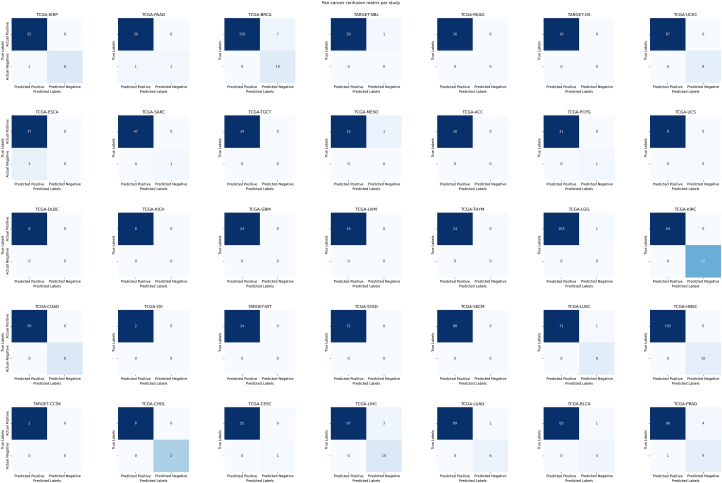
Detailed confusion matrix of CNN for each individual study present in the dataset.

## Appendix A Supplementary material

MMC 1 CNN training metrics for training set and validation set using Leaky Relu activation function when using pan-cancer dataset with all non-thyroid samples. MMC 1

MMC 2 CNN training metrics for training set and validation set using Standard Relu activation function when using pan-cancer dataset with all non-thyroid samples. MMC 2

MMC 3 CNN training metrics for training set and validation set using Standard Relu activation function when using pan-cancer dataset with non-thyroid solid-only samples. MMC 3
